# Healthcare workers’ perspectives on coronavirus testing availability: a cross sectional survey

**DOI:** 10.1186/s12913-021-06741-5

**Published:** 2021-07-21

**Authors:** Elena Byhoff, Jessica K. Paulus, Rubeen Guardado, Julia Zubiago, Alysse G. Wurcel

**Affiliations:** 1 Tufts Medical Center, 800 Washington Street Box #63, MA 02111 Boston, USA; 2 Tufts University School of Medicine, 145 Harrison Ave, MA 02111 Boston, USA

**Keywords:** Coronavirus, Healthcare workers, Disparities, Access to testing

## Abstract

**Background:**

Studies on the impact of the novel SARS-CoV-2 virus (COVID) for healthcare workers (HCWs) rarely include the full spectrum of hospital workers, including less visible patient support roles. In the early days of the pandemic, COVID testing was preferentially available to HCWs. The objective of this study was to understand how individual experiences for all HCWs during the pandemic were associated with perceptions of access to, and receipt of COVID testing .

**Methods:**

All hospital employees (*n* = 6736) in a single academic medical center in Boston, Massachusetts were invited to participate in a cross-sectional survey regarding perceived access to, and receipt of COVID testing during the first wave of the pandemic (March – August 2020). Responses were linked to human resources data. Log binomial univariate and multivariable models were used to estimate associations between individual and employment variables and COVID testing.

**Results:**

A total of 2543 employees responded to the survey (38 %). The mean age was 40 years (± 14). Respondents were female (76 %), white (55 %), worked as nurses (27 %), administrators (22 %) and patient support roles (22 %); 56 % of respondents wanted COVID testing. Age (RR 0.91, CI 0.88–0.93), full time status (RR 0.85, CI 0.79–0.92), employment tenure (RR 0.96, CI 0.94–0.98), changes in quality of life (RR 0.94, CI 0.91–0.96), changes in job duties (RR 1.19, CI 1.03–1.37), and worry about enough paid sick leave (RR 1.21, CI 1.12–1.30) were associated with interest in testing. Administrators (RR 0.64, CI 0.58–0.72) and patient support staff (RR 0.85, CI 0.78–0.92) were less likely than nurses to want testing. Age (RR 1.04, CI 1.01–1.07), material hardships (RR 0.87, CI 0.79–0.96), and employer sponsored insurance (RR 1.10, CI 1.00-1.22) were associated with receiving a COVID test. Among all employees, only administrative/facilities staff were less likely to receive COVID testing (RR 0.69, CI 0.59–0.79).

**Conclusions:**

This study adds to our understanding of how hospital employees view availability of COVID testing. Hazard pay or other supports for hospital workers may increase COVID testing rates. These findings may be applicable to perceived barriers towards vaccination receipt.

**Supplementary Information:**

The online version contains supplementary material available at 10.1186/s12913-021-06741-5.

## Background

Beginning in March 2020, The Centers for Disease Control (CDC) guidelines prioritized Health Care Workers (HCWs) for SARS-CoV-2 (COVID) testing. These guidelines were applied primarily to doctors and nurses leaving out other essential hospital workers including hourly-wage patient transporters, nursing assistants, translators, and food delivery workers who frequently come from minority communities.

To date, more than 300,000 HCWs have been infected with COVID in the U.S., and more than 1700 have died, with significant racial and ethnic disparities in infection and death rates [1–4]. Female essential workers also experience high risk of exposure due to disproportionate representation among frontline HCWs, and increased responsibilities in caring for children and elderly dependents.[5] Yet, a recent national HCW survey suggests that women are less likely to have been tested for COVID.[6] Geospatial evidence of decreased access to COVID testing in lower socioeconomic status communities is well documented, but there is little data on the structural and systematic barriers to COVID testing faced by minority communities.[7, 8] It is critical to understand differences in testing uptake for groups at highest risk for COVID infection and death—HCWs, specifically women and minority HCWs. The socioeconomic, racial, and ethnic diversity in HCWs represents a microcosm of the disparities in COVID impact nationwide.

In spring of 2020, Massachusetts had the 3rd highest positive COVID case rate in the U.S., with the Boston metropolitan area as the epicenter.[9] COVID testing offered to HCWs varied across the state, with some hospitals quickly establishing their own on-site testing, and others out-sourcing tests to commercial companies, academic institutions or the state Department of Public Health. On March 14, Tufts Medical Center (TMC)—a 415-bed academic medical center located in downtown Boston—was the first hospital in the region to provide on-site, no-cost COVID testing to all employees. All TMC employees were notified via email and web-based town halls that walk-in testing was available seven days a week in the hospital. CDC initial guidelines restricted testing to those with symptoms and known COVID contacts.[10] TMC testing capacity scaled up to offer testing to all employees regardless of symptoms.

Despite universal access to COVID testing, only 20 % of TMC employees have received COVID testing. This is worrisome in light of known COVID infection clusters in healthcare settings.[11–13] While on-site and no cost testing availability may have facilitated uptake, symptom and exposure screening, as well as mandatory reporting of employee’s department and manager may have created perceived barriers to testing. We conducted a survey to understand interest in COVID testing among HCWs during the pandemic. Our survey aimed to identify characteristics of HCWs who wanted COVID testing and who received testing. The survey instrument included items exploring HCWs’ perceptions of their workplace and individual or job-related factors that may have contributed to lower rates of COVID testing among HCWs during the pandemic surge.

## Methods

### Study settings and participants

All employees at TMC and the Physician’s Organization were considered HCWs for the purposes of this cross sectional study, and were invited to participate in a survey via email. Weekly and then daily reminder emails were sent to non-responders throughout the survey period to maximize response rates.[14] The study team met weekly with data specialists from Human Resources (HR) and Information Technology to ensure participation from all departments and hospital positions. Based on weekly survey response surveillance, members of the study team began in-person recruitment at locations throughout the hospital where there was limited access to computers or emails during the work day (environmental services breakrooms, food services preparation areas, equipment cleaning and sterilization spaces, maintenance rooms) or for departments that were unlikely to check their email due to heavy clinical workload (medical and surgical nursing floors, peri-operative suites, translator offices). Surveys were translated into 5 languages (Spanish, Haitian Creole, Portuguese, Simplified and Traditional Chinese). All respondents received a $10 gift card for their participation. This study was approved by the Tufts Medical Center Institutional Review Board.

### Survey development and data linkage

The survey was developed in consultation with national experts in survey design to evaluate social risks in health care settings and finalized by the study team, consisting of two physicians, two MPH level research associates, and an epidemiologist. Questions were drawn from validated questions in a repository of COVID related surveys to understand social determinants of health, financial hardships, mental health impacts, and perceived infection risk [15]. After expert review and feedback for content and validity, the survey was cognitively tested with employees at other Boston-area academic medical centers and field pretested with off-site IT employees to ensure appropriate timing, access, and flow of the survey. Unique survey links were generated by Qualtrics for all employees based on unique identifiers and then linked to detailed sociodemographic data provided by HR. Participants who completed in-person paper surveys were asked to provide their hospital login, which corresponded to their unique identifier. Beginning June 8, 2020 the hospital initiated a daily, mandatory COVID-19 symptom screen for all employees, which required knowledge and use of a hospital login. Symptom screens were meant to identify employees who potentially could expose co-workers and therefore should be COVID tested.[16] A positive screen resulted in a referral to occupational health for symptom and exposure review. Employees who did not regularly check or use workplace email were still able to provide their login for the in-person surveys. In person surveys were completed on paper, and manually entered and validated by the research team via double data entry.

#### Data collection

Using our survey and linked HR data, we created the following categories of variables to determine associations between HCWs’ individual characteristics and lived experiences and our primary outcomes:

### Socioeconomic, demographic and employment factors

HR provided employee data including age, gender, race, ethnicity, residence (city, state), job title, salary, receipt of employer sponsored insurance, hire date, full time equivalent (FTE) status, U.S. citizenship status, and marital/partnered status. Job titles were collapsed into the following categories: administrative (employees with no patient contact including research staff, finance, administrative support, clinic managers, human resources staff, billing staff, information technology and others); executive staff; facilities (plumbers, heating ventilation and air conditioning repair, electricians and other building or maintenance staff); nursing and nursing supports (registered nurses, medical assistants, licensed practical nurses, nurse practitioners, certified nurse anesthetists and others who work as nurses or directly support nursing staff and responsibilities); patient care (jobs with direct patient facing responsibilities that are neither nursing nor physicians – respiratory therapist, child life specialist, physical or occupational therapist, speech and language pathologist, social workers, and others); patient support (jobs that encounter patients regularly, but are not directly patient care – environmental services, interpreters, patient transporters, operating room technologists, dietary and hospitality services, pharmacists, clinical coordinators, security guards); and physicians (including attending physicians, resident and fellow physicians, and physician assistants).

Annual salary was calculated based on hourly wages for each job title. Non-physician employee annual salaries were calculated using hourly wage and based on full time (40) hours. Physician wages were provided as annual salary ranges instead of individual values owing to anonymity concerns. Physicians were then assigned salaries at the upper limit value, as physician annual salaries were notably higher than the non-physician annual salaries. Survey responses included number of household members and number of children in the household. Using both household number and annual salary, we calculated percentage of the Federal Poverty Level (%FPL) for each respondent. %FPL was categorized as ≤ 200, 201–400, 401–600, > 600.

### Employment changes and worries due to COVID

Survey respondents were asked about changes and stressors related to their work during the pandemic, including if they were furloughed or had hours cut, if and how their job responsibilities changed, how much they perceived their new job duties to be different from before the pandemic on a 5-point scale, concerns about using up Paid Time Off (PTO) or sick leave, if they have a second job, COVID related impacts to their second job, and changes in how they commute to work.

### Financial and social concerns related to COVID

Respondents were asked about any worries related to inability to afford living expenses including rent or mortgage, electricity or other utilities, food, medicine or medical bills, childcare or other paid caregivers, and travel to work. Respondents were also asked if they personally knew anyone who died or had been hospitalized due to COVID infection, or if anyone in their household had lost a job. All respondents were asked how they rated their quality of life before and during the pandemic on a 1–10 scale.

### Primary outcomes

The primary outcomes were (1) interest in and (2) receipt of COVID testing. Respondents were asked if they had ever wanted a COVID test since the start of the pandemic (March 2020). The survey specified COVID PCR testing as a nasal or oral swab, and not a blood test. See Appendix 1 for full survey.

### Statistical analysis

We describe the characteristics and survey responses of all respondents using summary statistics. We summarized the association between demographics, socioeconomic and workplace characteristics, employment/life changes related to the pandemic, and interest in testing using log binomial regression models to estimate univariate Risk Ratios (RR), their 95 % confidence intervals and associated p values. Among those respondents who indicated an interest in COVID testing, we applied the same approach to estimate associations between predictors and receipt of a COVID test. Collinearity between variables was examined using pairwise correlation coefficients. Where collinearity was identified, we selected variables for optimizing translational potential for developing either targeted outreach or employee support services. Multivariable models were built using candidate variables with statistically significant associations in univariate analysis (*p* < 0.05) for each outcome of interest: (1) wanting a COVID test and (2) receiving a test. Race was decided *a priori* to be included in both multivariable models based on extant literature highlighting racial disparities in COVID testing.[17–19] All analyses were performed using STATA 15.

## Results

**Fig. 1:**
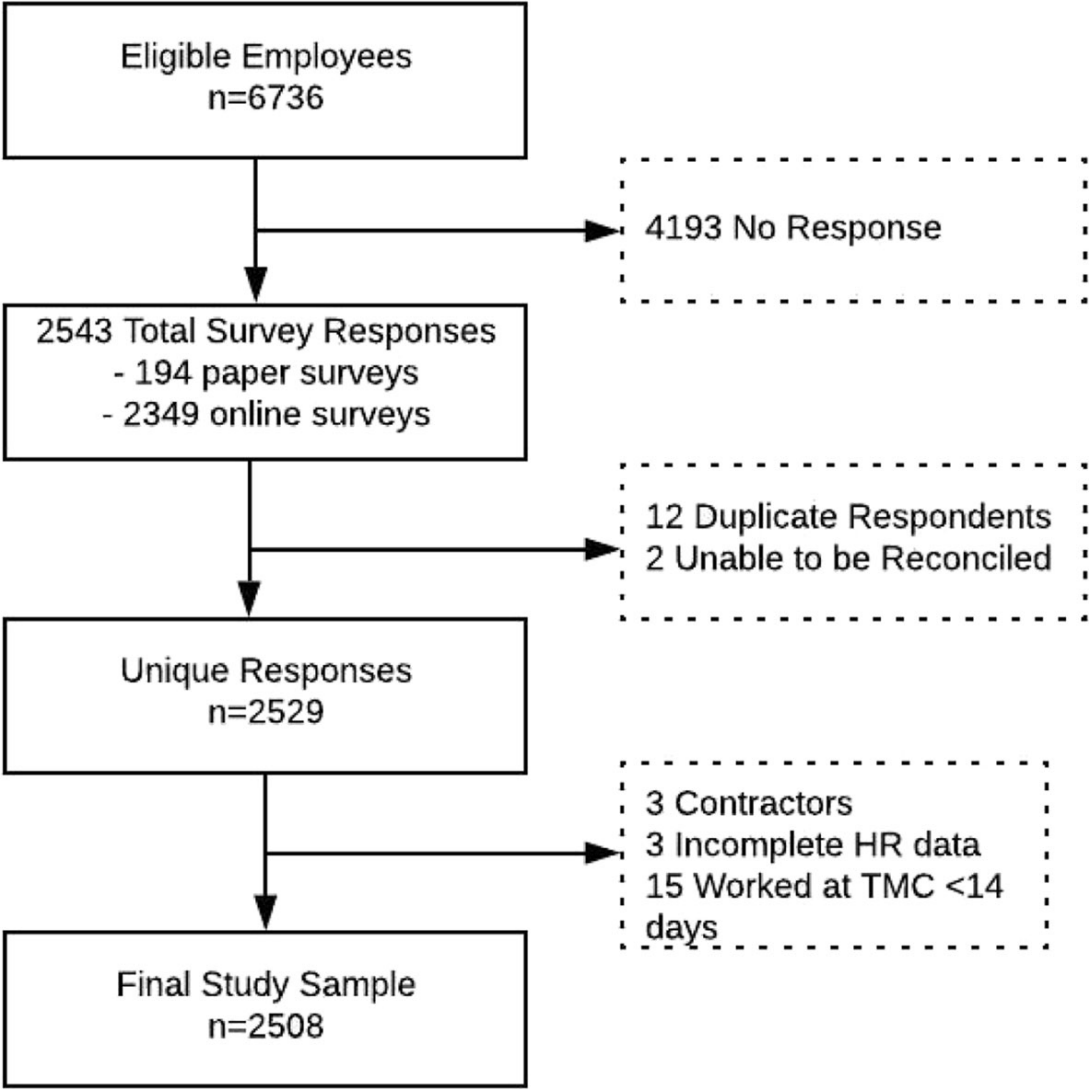
Sampling and participation of hospital employees in a COVID testing survey

Survey data were collected from July 9 – August 14, 2020. Of 6736 eligible employees, 2543 surveys were completed (38 % response rate). Our final analytic sample included 2501 unique respondents with linked HR data (see Fig. 1).

Highlights of the study population characteristics are shown in Table 1, with full details in Appendix 2. The mean age of respondents was 40 years (± 14), and were predominantly female (76 %) and white (55 %). Respondents represented all job categories reported by HR, with nursing (27 %), administrative (22 %) and patient support (22 %) as the largest subgroups. The median annual salary of respondents was $74,797 (mean $97,142; IQR $51,813 − 129,418), and 78 % were full-time employees. 17 % of respondents live at or below 200 % FPL, 28 % expressed worry related to finances, and 19 % were worried about affording next month’s rent or mortgage. Respondents felt their self-perceived quality of life got worse by a mean of 1.9 points during the pandemic, 56 % reported their job is more stressful, 91 % reported changes in their job responsibilities, and 54 % reported being worried about using up sick time/PTO.

**Table 1:** Select Demographic and Employment Characteristics of Survey Respondents

	Total *N* = 2,508
**Age in years, mean (SD, Range)**	40 (± 14, 19–81)
**Age Categories in years, (%)**	
19–29	735 (29)
30–39	689 (28)
40–49	357 (14)
50–59	434 (17)
≥ 60	293 (12)
**Gender (%)**	
Female	1,895 (76)
**Race/Ethnicity (%)**	
white	1,394 (55)
Black	185 (7)
Hispanic or Latino	118 (5)
Asian	306 (12)
American Indian/Alaskan Native	6 (< 1)
Two or More	66 (3)
Not Applicable (Non-US)	3 (< 1)
Missing	430 (17)
**Marital Status (%)**	***N* = 1,893**
Single	1,257 (66)
Married	634 (33)
Other (Partnership/ Divorced/Widowed)	7 (< 1)
**Financial Concerns**	
**Worried about NOT having enough money for**:	
next month’s rent (%)	480 (20)
next month’s utilities (%)	294 (12)
food (%)	257 (10)
medical bills (%)	230 (9)
childcare or care of a loved one (%)	212 (8)
transportation to work (%)	187 (7)
**Reported any worry related to finances (%)**	
Yes	705 (28)
**Federal Poverty Level (%)**	***N* = 2,490**
≤ 200 %	417 (17)
201-400 %	838 (34)
401-600 %	595 (24)
> 600 %	640 (26)
Occupational & Quality of Life Changes	
**Annual Salary in USD, mean (SD; Range)**	98,142 (± 72,973; 31,054 − 1,088,269)
**Annual Salary (median; IQR)**	(74,797; 51,813 − 129,418)
**Full-Time/Part-Time Employment (%)**	
Part-Time	543 (22)
Full-Time	1,965 (78)
**Years at Tufts Medical Center, mean (SD)**	8 (± 10)
**Job Category (%)**	
Admin (Research, Finance)	548 (22)
Executives	43 (2)
Facilities	88 (3)
Nursing/Nursing Support	675 (27)
Patient Care (RT^a^, PT/OT^b^, SLP^c^)	227 (9)
Patient Support (Pharmacist, Dietary, EVS^d^)	550 (22)
Physicians	377 (15)
**Self-perceived quality of life pre-COVID pandemic, mean (SD)**	7.5 (± 1.7)
**Self-perceived quality of life during COVID pandemic, mean (SD)**	5.6 (± 1.9)
**In what ways has your job changed**:	
I have been furloughed (%)	160 (6)
I have reduced hours (%)	55 (2)
Hours have increased (%)	418 (17)
I have been asked to work in a different place (%)	750 (30)
Asked to work from home (%)	689 (27)
I am on leave of absence (%)	15 (< 1)
My job is more stressful (%)	1,394 (56)
**Reported any changes to job**	
Yes	2,280 (91)
**How much has employee’s job changed (%)**	***N* = 2,506**
Not at all	154 (6)
Somewhat	699 (28)
A good deal	734 (29)
Very much	869 (35)
Don’t know	50 (2)
**Worried about using up sick leave/PTO**^e^**(%)**	***N* = 2,464**
Not at all	980 (40)
Somewhat	660 (27)
A good deal	271 (11)
Very much	403 (16)
I don’t get sick leave or paid time off	150 (6)

^a^ Respiratory Therapist

^b^ Physical Therapist/ Occupational Therapist

^c^ Speech and Language Pathologist

^d^ Environmental Services

^e^ Paid Time Off

### Interest in COVID testing

Of all respondents, 56 % (*n* = 1396) reported interest in COVID testing (Table 2). Factors associated with not wanting testing included age ≥ 60 years (RR 0.70), full time employment (RR 0.85), employees who worked 6–10 years (RR 0.75) and > 10 years (RR 0.81) compared with those working < 1 year, administrative/facilities staff and patient care/support staff (RR 0.64 and RR 0.85) compared with nursing staff. Reporting any changes to job duties (RR 1.19), magnitude of job changes in response to the pandemic (RR 1.40), and worry related to use of sick time/PTO was associated with greater interest in testing (RR 1.21). The multivariable model included age, race (white vs. non-white), change in perceived quality of life, part-time status, job category, and magnitude of job change. Multivariable model results showed persistent associations and directionality between reporting wanting COVID testing and independent variables. Worry about using up sick leave (RR 1.17) and larger perceived changes in job responsibility (RR1.24) both had the highest risk ratio of wanting COVID testing during the pandemic.

**Table 2:** Characteristics of Respondents who wanted COVID testing – Univariate and Multivariable Models

	Wanted COVID-19 Test? (***N***=2501)		Univariate			Multivariable		
	No (***n***=1,105)	Yes (***n***=1,396)	RR	95% CI	***P***-value	RR	95% CI	***P***-value
**Age in years, mean (SD)**	42.31 (13.62)	38.31 (13.39)	0.91	.88, 93	P< 0.001	0.93	.90, .95	P < 0.001
**Gender (%)**								
Male	286 (47)	322 (53)	Ref	-	0.11			
Female	819 (43)	1,074 (57)	1.07	.98, 1.17				
**Race/Ethnicity (%)**								
white	599 (43)	795 (57)	Ref	-	0.03	Ref	-	0.03
Non-white	327 (48)	351 (52)	0.91	.83, .99		0.92	.85, 1.00	
Missing	179 (42)	250 (58)	1.02	.93, 1.12		1.05	.96, 1.14	
**Massachusetts Resident (%)**								
No	42 (40)	64 (60)	Ref	-	0.31			
Yes	1,063 (44)	1,332 (56)	0.92	.79, 1.08				
**Any use of public transportation to go to work during pandemic (%)**	***N*=1,085**	***N*=1,354**						
No	873 (44)	1,098 (56)	Ref	-	0.70			
Yes	212 (45)	256 (55)	0.98	.90, 1.08				
**Living with others in household (%)**	***N*=1,094**	***N*=1,393**						
No	115 (40)	176 (60)	Ref	-	0.09			
Yes	979 (45)	1,217 (55)	0.92	.83, 1.01				
**Marital Status (%)**	***N*=877**	***N*=1,016**						
Single	559 (45)	693 (55)	Ref	-	0.06			
Married	316 (50)	318 (50)	0.91	.83, .99				
Other (Partnered/ Divorced/Widowed)	2 (29)	5 (71)	1.29	.81, 2.07				
**Any worry related to finances (%)**								
No	814 (45)	986 (55)	Ref	**-**	0.09			
Yes	291 (42)	410 (58)	1.07	.99, 1.15				
**Change in perceived quality of life (SD)**	-1.66 (2.21)	-2.04 (2.25)	0.94	.91, .96	P < 0.001	0.95	.92, .98	0.00
**Salary Categories in USD (%)**	***N*=1,102**	***N*=1,395**						
≤ $50,000	265 (45)	323 (55)	Ref	-	0.70			
$50,001 - $75,000	296 (43)	393 (57)	1.04	.94, 1.15				
$75,001 - $100,000	155 (41)	219 (59)	1.07	.95, 1.19				
$100,001 - $150,000	220 (46)	261 (54)	0.99	.88, 1.10				
$150,001 - $200,000	106 (46)	123 (54)	0.98	.85, 1.13				
> $200,000	60 (44)	76 (56)	1.02	.86, 1.20				
**Full-Time/Part-Time Employment (%)**								
Part-Time	199 (37)	344 (63)	Ref	-	P < 0.001	Ref	-	0.00
Full-Time	906 (46)	1,052 (54)	0.85	.79, .92		0.96	.88, 1.04	
**Years at Tufts Medical Center (%)**								
< 1 year	175 (38)	280 (62)	Ref	-	P < 0.001			
1-5 year	468 (41)	677 (59)	0.96	0.88, 1.05				
6-10 years	138 (54)	119 (46)	0.75	0.65, 0.87				
> 10 years	324 (50)	320 (50)	0.81	0.73, 0.90				
**Job Category (%)**								
Nursing/Nursing Support	232 (34)	443 (66)	Ref	-	P < 0.001	Ref	-	P < 0.000
Admin (Research, Finance)/Facilities	364 (58)	267 (42)	0.64	.58, .72		0.74	.66, .83	
Executive	21 (49)	22 (51)	0.78	.58, 1.05		0.95	.69, 1.30	
Patient Care (RT^a^, PT/OT^b^, SLP^c^)/Patient Support (Pharmacy, Dietary, EVS^d^)	345 (45)	430 (55)	0.85	.78, .92		0.89	.82, .98	
Physicians	143 (38)	234 (62)	0.95	.86, 1.04		1.07	.96, 1.19	
**Employer Sponsored Insurance (%)**	***N*=1,098**	***N*=1,389**						
No	271 (42)	373 (58)	Ref	**-**	0.21			
Yes	827 (45)	1,016 (55)	0.95	.88, 1.03				
**How much has employee's job changed (%)**	***N*= 1,104**	***N*=1,395**						
Not at all/Don’t know	116 (57)	87 (43)	Ref	-	P < 0.001	Ref	-	P < 0.001
Somewhat	345 (50)	349 (50)	1.17	.98, 1.40		1.14	.95, 1.36	
A good deal/Very much	643 (40)	959 (60)	1.40	1.19, 1.65		1.24	1.05, 1.47	
**Does employee have second job (%)**	***N*=1,104**	***N*=1,395**						
No	950 (45)	1,165 (55)	Ref	**-**	0.07			
Yes	154 (40)	230 (60)	1.09	.99, 1.19				
**Worried about using up sick leave/PTO**^e^ **(%)**	***N*=1,085**	***N*=1,374**						
No	560 (50)	566 (50)	Ref	-	P < 0.001	Ref	-	P < 0.001
Yes	525 (39)	808 (61)	1.21	1.12, 1.30		1.17	1.09, 1.26	

^a^Respiratory Therapist

^b^Physical Therapist/ Occupational Therapist

^c^Speech and Language Pathologist

^d^Environmental Services

^e^Paid Time Off

### Received COVID testing

Of the 1396 respondents who wanted COVID testing, 873 (63 %) received testing (Table 3). In the univariate analyses, every 10 year increase in age was associated with a 4% increased likelihood of receiving a COVID test (RR 1.04). Reporting any financial worries was associated with decreased likelihood of receiving a COVID test (RR 0.87). The highest salary and %FPL were associated with increased likelihood of COVID testing compared to the lowest earners. Receiving employer sponsored insurance (RR 1.10) was associated with increased likelihood of receiving a COVID test. Among all HCWs, only non-patient facing job categories (administrative/facilities) were less likely to receive COVID testing than the reference of nursing staff (RR 0.69). The multivariable model included age, race (white vs. non-white), worries related to finances, job category, enrollment in employer sponsored health insurance, and magnitude of job change. In the multivariable model, financial worries, having employer sponsored insurance and magnitude of job changes lost significance, while age and job category remained significantly associated with receiving a COVID test. Race was not significantly associated with receiving a COVID test in either model.

**Table 3:** Characteristics of Respondents who received COVID Testing – Univariate and Multivariable Models

	Received COVID-19 Test? (***N***=1,395)		Univariate			Multivariable		
	No (***N***=522)	Yes (***N***=873)	RR	95% CI	***P***-Value	RR	95% CI	***P***-Value
**Age in years, mean (SD)**	37 (13)	39 (13)	1.04	1.01, 1.07	0.01	1.03	1.00, 1.06	0.03
**Gender (%)**								
Male	110 (34)	211 (66)	Ref	-	0.17			
Female	412 (38)	662 (62)	0.94	.86, 1.03				
**Race/Ethnicity (%)**								
white	281 (35)	514 (65)	Ref	-	0.63	Ref	-	0.80
Non-white	129 (37)	221 (63)	0.98	.89, 1.07		1.00	.91, 1.10	
Missing	112 (45)	138 (55)	0.85	.76, .97		0.96	.84, 1.09	
**Massachusetts Resident (%)**								
No	26 (41)	38 (59)	Ref	-	0.60			
Yes	496 (37)	835 (63)	1.06	.86, 1.30				
**Any use of public transportation to go to work during pandemic (%)**	***N*=505**	***N*=848**						
No	404 (37)	694 (63)	Ref	-	0.41			
Yes	101 (40)	154 (60)	0.96	.86, 1.07				
**Living with others in household (%)**	***N*=522**	***N*=807**						
No	66 (38)	110 (63)	Ref	-	1.00			
Yes	456 (38)	760 (63	1.00	.88, 1.13				
**Marital Status (%)**	***N*=377**	***N*=638**						
Single	256 (37)	436 (63)	Ref	-	0.68			
Married	117 (37)	201 (63)	1.00	.91, 1.11				
Other (Partnered/Divorced/Widowed)	4 (80)	1 (20)	0.32	.05, 1.83				
**Any worry related to finances (%**								
No	345 (35)	640 (65)	Ref	**-**	0.01	Ref	-	0.05
Yes	177 (43)	233 (57)	0.87	.79, 96		0.93	.84, 1.02	
**Change in perceived quality of life (SD)**	-2.03 (2.17)	-2.05 (2.30)	1.00	.96, 1.04	0.89			
**Salary Categories in USD (%)**	***N*=521**	***N*=873**						
≤ $50,000	124 (39)	198 (61)	Ref	-	0.04			
$50,001 - $75,000	151 (38)	242 (62)	1.00	.89, 1.13				
$75,001 - $100,000	90 (41)	129 (59)	0.96	.83, .1.10				
$100,001 - $150,000	98 (38)	163 (62)	1.02	.89, 1.15				
$150,001 - $200,000	37 (30)	86 (70)	1.14	.98, 1.31				
> $200,000	21 (28)	55 (72)	1.18	1.00, 1.39				
**Full-Time/Part-Time Employment (%)**								
Part-Time	130 (38)	214 (62)	Ref	-	0.87			
Full-Time	392 (37)	659 (63)	1.01	.92, 1.11				
**Years at Tufts Medical Center (%)**								
< 1 year	133 (48)	147 (53)	Ref	-	< 0.001			
1-5 year	253 (37)	423 (63)	1.19	1.05, 1.35				
6-10 years	40 (34)	79 (66)	1.26	1.07, 1.50				
> 10 years	96 (30)	224 (70)	1.33	1.17, 1.52				
**Job Category (%)**								
Nursing/Nursing Support	232 (34)	443 (66)	Ref	-	< 0.001	Ref	-	< 0.001
Admin (Research, Finance)/Facilities	364 (58)	267 (42)	0.69	.59, .79		0.70	.60, .81	
Executive	21 (49)	22 (51)	1.08	.83, 1.41		0.98	.74, 1.30	
Patient Care (RT^a^, PT/OT^b^, SLP^c^)/Patient Support (Pharmacist, Dietary, EVS^d^)	345 (45)	430 (55)	0.92	.84, 1.02		0.95	.86, 1.05	
Physicians	143 (38)	234 (63)	1.07	.97, 1.19		1.08	.97, 1.20	
**Employer Sponsored Insurance (%)**	***N*=519**	***N*=869**						
No	156 (42)	217 (58)	Ref	**-**	0.05	Ref	-	0.41
Yes	363 (36)	652 (64)	1.10	1.00, 1.22		1.04	.95, 1.15	
**How much has employee's job changed (%)**	***N*=522**	***N*=872**						
Not at all/Don’t know	35 (40)	52 (60)	Ref	-	0.05	Ref	-	0.10
Somewhat	148 (42)	201 (58)	0.96	.79, 1.17		0.92	.77, 1.12	
A good deal/Very much	339 (35)	619 (65)	1.08	.90, 1.29		1.01	.84, 1.20	
**Does employee have second job (%)**	***N*=522**	***N*=872**						
No	436 (37)	729 (63)	Ref	**-**	0.97			
Yes	86 (38)	143 (62)	1.00	.89, 1.11				
**Worried about using up sick leave/PTO**^e^ **(%)**	***N*=512**	***N*=861**						
Not at all/I don't get sick leave/paid time off	203 (36)	363 (64)	Ref	-	0.69			
Somewhat	147 (40)	220 (60)	0.93	.84, 1.04				
A good deal/Very much	162 (37)	278 (63)	0.99	.90, 1.08				

^a^Respiratory Therapist

^b^Physical Therapist/ Occupational Therapist

^c^ Speech and Language Pathologist

^d^Environmental Services

^e^Paid Time Off

## Discussion

Understanding how to identify HCWs who may be at risk for COVID, but not interested in or able to access COVID testing is of critical importance. Our study found key differences between HCWs who wanted COVID testing, and those who received COVID testing. HCWs who wanted testing were more likely to be younger, white, working part-time, a nurse or doctor, worried about using up paid sick time, and felt that their job responsibilities have changed substantially during the pandemic. Developing communication and outreach protocols by the health care system to increase interest in testing could use these characteristics to target HCWs in patient care and support roles, in particular those who are older and non-white. Increasing interest in COVID testing is a critical first step in to implement broader COVID testing in health care settings.

It is important to understand HCWs’ individual socioeconomic, demographic, and employment characteristics, perceptions of stress and job changes, to understand HCWs’ interest in and receipt of COVID testing. Prior work has used either individually reported job characteristics with limited socioeconomic data or administrative data to estimate population-wide characteristics.[6, 7, 20, 21] Our study demonstrates clear socioeconomic disparities and financial hardships facing HCWs during the pandemic, with 28 % of respondents reporting at least one financial concern, 20 % worry about affording next month’s rent, and 17 % living at or below 200 % of FPL. Socioeconomic factors were not significantly associated with interest in COVID testing, but were significantly associated with receipt of COVID testing. Proponents of extended paid sick leave or hazard pay or increasing wages for frontline essential HCWs may be a reasonable strategy to encourage equitable receipt of COVID testing.[22, 23].

Several sociodemographic factors identified as key drivers of disparities in the COVID-19 pandemic in prior studies were not associated with COVID testing in our sample.[17–19] Despite a strong effort to recruit HCWs from all socioeconomic groups and all job categories, we learned during the course of data collection that some of the most vulnerable workers in terms of income, non-English language, and high exposure risk jobs (cleaning services, nutritional services, dialysis services) were not employed by the hospital, but rather as contractors. Due to the nature of contracted labor, these workers did not meet study inclusion criteria because there was no HR data available for linkage. Many sectors, including health care, increasingly rely on outsourcing labor to reduce costs and grow profit margins.[24, 25] Labor economists posit that increasing this “shadow workforce” has led to widening socioeconomic inequalities due to loss of workplace protections for contracted workers with the potential for exacerbation of inequities during the pandemic.[26–30] Contracted labor is found more frequently among low-skilled and low-income workers, and the pandemic has forced difficult choices between wages and safety.[3, 31, 32] Future work to evaluate HCWs access to and receipt of COVID testing must also include contractors who work on the fringes of health care systems, employed by multinational contracting firms who may not readily offer workplace protections and essential benefits similar to hospital employees on the frontlines of the pandemic.

Early identification is a critical component of the test-trace-isolate public health response.[33–35] Beyond individual and workplace factors, national testing supply shortages, failure of a cohesive national testing policy, and changing testing guidelines, among others, have resulted in perceived and experienced difficulties in COVID testing, even among HCWs.[36–40] Despite broad access to testing, our study found only 55 % of respondents reported that they wanted COVID testing. As many states and health care systems are left to determine testing protocols on their own, this study sheds an important light on the distinct groups of HCWs who are and are not being tested for COVID. The CDC’s ongoing surveillance of HCW rates of COVID infection and death have demonstrated that patient care, particularly among nurses, is one of the biggest risk factors in HCW infection.[41] We found that doctors and other patient-facing positions are being tested at equal rates as our nursing staff. Employees earning the highest wages are more likely to receive testing than the lowest wage group, likely representing high rates of testing among doctors and nurses compared to other HCWs.

This study has several limitations of note. First, it was conducted at a single academic medical center that located in an early COVID epicenter. CDC testing guidelines were rapidly changing in the early pandemic months due to supply chain constraints, and HCW perspectives in an early hotspot on testing may have been biased by wide media coverage of testing shortages despite workplace testing availability and may not represent locations with later surges. While the study team engaged in multiple modalities of recruitment to ensure the widest possible representation from all HCW job descriptions, 96 % of our respondents took the survey in English and may not represent the linguistic diversity of HCWs generally. Missing data on race and ethnicity (17 %) limited our power to detect racial and ethnic differences for our primary outcomes. It is possible that selection bias contributed to non-response among some hospital employees.

## Conclusions

While hospital leadership created near universal access to COVID-19 testing during the first wave of the pandemic, only half of all hospital employees reported wanting COVID-19 testing. Hospital and workplace policies can be constructed to address disparities in COVID-19 testing due to financial concerns, including provision of extended sick leave policies. Review of employment policy, including hazard pay and paid time off are necessary, but may not be sufficient, to increase HCW interest in COVID-19 testing. As public health focus shifts from emphasis on COVID testing to COVID vaccination, it is imperative to understand how workplace policies may affect interest in, and uptake of, vaccination among HCWs.

## Availability of data and materials

The datasets generated and analyzed during the current study are not publicly available due to protected employment files, but anonymized survey results are available from the corresponding author on reasonable request.

## Supplementary Information

**Additional file 1.**

**Additional file 2.**

## Data Availability

Availability of de-identified survey data is available upon request.
