# Supplementary material for: Healthcare workers’ perspectives on coronavirus testing availability: a cross sectional survey
Source: BMC Health Serv Res. 2021 Jul 21;21:719. doi: 10.1186/s12913-021-06741-5 (PMC8294832; doi:10.1186/s12913-021-06741-5)
Supplement: Supplementary file 1 — Additional file 1. [file 12913_2021_6741_MOESM1_ESM.pdf]

COVID-19 Employee Survey

CONSENT INTRO

Instructions: Unless otherwise specified, please select only one answer for each question. If you are uncertain, or choose not to answer, leave the question blank.

(1) What is your main form of transportation to work since March 2020 (the start of the COVID pandemic)?

01. Car (drive alone)
02. Car (carpool with other people)
03. Public Transit
04. Bicycle
05. Walk
06. Uber/Lyft/Taxi or other rideshare
07. N/A, working from home
08. Other : [Please, specify.]

(2) Is this different from your usual transportation before the COVID-19 pandemic?

00. No
01. Yes

(2A) IF YES, what was your transportation before the COVID-19 pandemic?

01. Car (drive alone)
02. Car (carpool with other people)
03. Public Transit
04. Bicycle
05. Walk
06. Uber
07. Other: [Please, specify.]

(3) How much has your job at Tufts Medical Center been affected by the COVID-19 pandemic?

01. Not at all
02. Somewhat
03. A good deal
04. Very much
05. Don’t know

(4) In what ways has your job been affected? *Check all that apply.*

01. I have been furloughed through reduced working hours (communicated to me through my manager)
02. I have reduced my hours because of other obligations
03. I have had my work hours increased
04. I have been asked to work in a different place than usual
05. I have been asked to work from home
06. I am on leave of absence
07. My work is more stressful
08. None of these ways
09. Other: Please, specify.

(5) How worried are you about using up your sick leave and paid time off?

01. Not at all
02. Somewhat
03. A good deal
04. Very much
05. I don’t get sick leave or paid time off

(6) Do you have any other jobs outside of your job at Tufts Medical Center? This includes work as an employee for another company, an independent contractor (Uber, Lyft driver, moonlighter), or any other way you earn money (babysitting, house cleaning, etc.).

00. No
01. Yes

(6A) IF YES, how has your other employment been affected by the COVID-19 pandemic (after March 2020)?

01. I am still going to my other job/workplace for the same number of hours as before the pandemic
02. I am still going to my other workplace but am working reduced hours
03. I am still going to my other workplace and am working more hours
04. I am working from home for my other employment
05. I lost my other job
06. I had to quit my other job because I need to take care of people who depend on me (children, parents)

(7A) Imagine a ladder with steps numbered from zero at the bottom to 10 at the top. The top of the ladder represents the best possible life for you and the bottom of the ladder represents the worst possible life for you. Where on this ladder would you put yourself before March 1, 2020 (the start of the COVID-19 pandemic)? 1 is the bottom and 10 is the top.

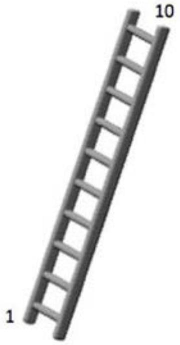

01	02	03	04	05	06	07	08	09	10

(7B) Since March 2020 (the start of the COVID-19 pandemic), where would you place yourself now? 1 is the bottom and 10 is the top.

01	02	03	04	05	06	07	08	09	10

(8) Since March 2020 (the start of the COVID-19 pandemic), have you been worried about any of the following? *Check all that apply.*

01. Not having enough money to pay next month's rent or mortgage
02. Not having enough money to pay next month's heating, electricity, or internet bills
03. Not having enough money to buy food
04. Not having enough money to pay for medicine or medical bills
05. Not having enough money to pay for childcare or other care for a family member or loved one
06. Not having enough money to pay for transportation to work

(9) How many people live in your house (include yourself)?

01	02	03	04	05	06	07	08	09	10+

(10) How many people that live in your home are under 18 years old?

00	01	02	03	04	05	06	07	08	09	10+

(11) How many people use/share your kitchen or living room (include yourself)?

01	02	03	04	05	06	07	08	09	10

(12) Did anyone who lives in your house lose their job?

00. No
01. Yes

(13) How likely do you think it is that you will get COVID-19 in the next month?

01. Very likely
02. Somewhat likely
03. Neither likely nor unlikely
04. Somewhat unlikely
05. Very unlikely

(14) Has anyone you know (e.g., family members, friends, neighbors, coworkers) died or been hospitalized from COVID-19?

00. No
01. Yes

In the following questions we are referring to the nasal or oral swab test for COVID-19 (<u>not the blood test</u>):

(15) Since the start of the COVID-19 pandemic (March, 2020), did you want to get a COVID-19 test?

00. No. **-→SKIP TO 19**
01. Yes**---> Continue to #16**

(16) Did you want a test for any of these reasons? ***Check all that apply.***

01. I had a direct exposure (I was physically close to someone who I know tested positive for COVID-19)
01. I was told I needed to get tested to return to work
02. I heard that someone I knew had COVID-19
03. I had symptoms
04. I was worried about infecting others
05. Something else:

Please, specify.

(17) Did you try to get a test in any of the following ways? ***Check all that apply.***

01. Visit Tufts Medical Center (Tye Pavilion or Josiah Quincy) testing center?
02. Go to another COVID testing site?
03. Call your doctor for a test?

04. Call employee health/occupational health for a test?
05. Go to an Emergency Room for a test?
06. Go to an urgent care for a test?

(18) Were you tested for COVID-19?

00. No
01. Yes

(18YA) IF YES, how many times have you been tested for COVID-19?

01	02	03	04	05	06	07	08	09	10

(18YB) IF YES, have you ever tested positive for COVID-19?

00. No
01. Yes **-----> *IF YES, Go to PCR positive questions (TUSM)***

(19) Why did you not get a COVID-19 test? *Check all that apply.*

01. Worried about lost wages
02. Worried about using sick time or PTO (paid time off)
03. Worried about what people would think of me if I tested positive.
04. Could not get childcare
05. Worried about cost of testing
06. Did not meet testing criteria
07. Could not get transportation to testing site
08. Worried about my family if I had to quarantine
09. Worried about the test hurting
10. Testing center not open when I needed it
11. Other: Please, specify.

(20) We will ask some people to participate in another research study to test for COVID antibodies (blood test). Can we contact you?

00. No
01. Yes

(21) Can we contact you in the future for additional COVID-19 research studies?

00. No

01. Yes

End of Survey - Thank you!
