# Supplementary material for: Healthcare workers’ perspectives on coronavirus testing availability: a cross sectional survey
Source: BMC Health Serv Res. 2021 Jul 21;21:719. doi: 10.1186/s12913-021-06741-5 (PMC8294832; doi:10.1186/s12913-021-06741-5)
Supplement: Supplementary file 2 — Additional file 2. [file 12913_2021_6741_MOESM2_ESM.docx]

**Appendix Table 1**: Full Respondent Characteristics

|  | **Total N=2,508** |
| --- | --- |
| **Age in years, mean (SD, Range)** | 40 (± 14, 19 - 81) |
| **Age Categories in years, (%)** | |
| 19-29 | 735 (29) |
| 30-39 | 689 (28) |
| 40-49 | 357 (14) |
| 50-59 | 434 (17) |
| ≥ 60 | 293 (12) |
| **Gender (%)** | |
| Female | 1,895 (76) |
| **Race/Ethnicity (%)** | |
| white | 1,394 (55) |
| Black | 185 (7) |
| Hispanic or Latino | 118 (5) |
| Asian | 306 (12) |
| American Indian/Alaskan Native | 6 (<1) |
| Two or More | 66 (3) |
| Not Applicable (Non-US) | 3 (<1) |
| Missing | 430 (17) |
| **Survey Language Selected (%)** | |
| English | 2,450 (98) |
| **Massachusetts Resident (%)** | |
| Yes | 2,402 (96) |
|  |  |
| **Employee's Home Town Case Rate on August 20th (%)** | **N=2,408** |
| <5 cases in the last 14-days | 357 (15) |
| <4 avg. daily cases per 100,000 people | 945 (39) |
| 4-8 avg. daily cases per 100,000 people | 927 (39) |
| >8 avg. daily cases per 100,000 people | 179 (7) |
|  |  |
| **Living with others in household (%)** | **N=2,493** |
| Yes | 2,199 (88) |
|  |  |
| **Children in household (%)** | **N=2,490** |
| Yes | 852 (34) |
|  |  |
| **Marital Status (%)** | **N=1,893** |
| Single | 1,257 (66) |
| Married | 634 (33) |
| Other (Partnership/ Divorced/Widowed) | 7 (<1) |
| Financial Concerns | |
| **Worried about NOT having enough money for:** | |
| next month's rent (%) | 480 (20) |
| next month's utilities (%) | 294 (12) |
| food (%) | 257 (10) |
| medical bills (%) | 230 (9) |
| childcare or care of a loved one (%) | 212 (8) |
| transportation to work (%) | 187 (7) |
| **Reported any worry related to finances (%)** | |
| Yes | 705 (28) |
|  |  |
| **Federal Poverty Level (%)** | **N=2,490** |
| ≤ 200% | 417 (17) |
| 201%-400% | 838 (34) |
| 401%-600% | 595 (24) |
| > 600% | 640 (26) |
|  |  |
| Occupational & Quality of Life Changes | |
| **Annual Salary in USD, mean (SD; Range)** | 98,142  ( ± 72,973; 31,054 - 1,088,269) |
| **(median; IQR)** | (74,797; 51,813 - 129,418) |
| **Salary Categories in USD (%)** | **N=2,504** |
| ≤ $50,000 | 593 (24) |
| $50,001 - $75,000 | 691 (28) |
| $75,001 - $100,000 | 374 (15) |
| $100,001 - $150,000 | 481 (19) |
| $150,001 - $200,000 | 229 (9) |
| > $200,000 | 136 (5) |
| **Salary/Hourly Wage Employee (%)** | |
| Salary | 1,150 (46) |
| Hourly | 1,358 (54) |
| **Full-Time/Part-Time Employment (%)** | |
| Part-Time | 543 (22) |
| Full-Time | 1,965 (78) |
| **FTE (%)** |  |
| < .5 | 19 (1) |
| .5 - .99 | 524 (21) |
| 1.0 | 1,965 (78) |
| **Years at Tufts Medical Center, mean (SD)** | 8 (±10) |
| **Years at Tufts Medical Center (%)** | |
| < 1 year | 455 (18) |
| 1-5 year | 1,147 (46) |
| 6-10 years | 258 (10) |
| > 10 years | 648 (26) |
| **Job Category (%)** | |
| Admin (Research, Finance) | 548 (22) |
| Executives | 43 (2) |
| Facilities | 88 (3) |
| Nursing/Nursing Support | 675 (27) |
| Patient Care (resp therapist, PT/OT, SLP) | 227 (9) |
| Patient Support (Pharm, Dietary, EVS) | 550 (22) |
| Physicians | 377 (15) |
|  |  |
| **Insurance Through Tufts Medical Center (%)** | **N=2,494** |
| Yes | 1,848 (74) |
|  |  |
| **Self perceived quality of life pre-COVID pandemic, mean (SD)** | 7.5 (± 1.7) |
| **Self perceived quality of life during COVID pandemic, mean (SD)** | 5.6 (± 1.9) |
|  |  |
| **Change in Transportation to work due to COVID-19 (%)** | **N=2,505** |
| Yes | 1,042 (42) |
|  |  |
| **Any use of public transportation to go to work during pandemic (%)** | **N=2,446** |
| Yes | 474 (19) |
|  |  |
| **In what ways has your job changed:** | |
| I have been furloughed (%) | 160 (6) |
| I have reduced hours (%) | 55 (2) |
| Hours have increased (%) | 418 (17) |
| I have been asked to work in a different place (%) | 750 (30) |
| Asked to work from home (%) | 689 (27) |
| I am on leave of absence (%) | 15 (<1) |
| My job is more stressful (%) | 1,394 (56) |
|  |  |
| **Reported any changes to job** | |
| Yes | 2,280 (91) |
| **How much has employee's job changed (%)** | **N=2,506** |
| Not at all | 154 (6) |
| Somewhat | 699 (28) |
| A good deal | 734 (29) |
| Very much | 869 (35) |
| Don't know | 50 (2) |
|  |  |
| **Does employee have second job (%)** | **N=2,506** |
| Yes | 385 (15) |
|  |  |
| **Worried about using up sick leave/paid time off (%)** | **N=2,464** |
| Not at all | 980 (40) |
| Somewhat | 660 (27) |
| A good deal | 271 (11) |
| Very much | 403 (16) |
| I don't get sick leave or paid time off | 150 (6) |
